# Short-term effects of roxadustat on serum copper and iron changes in a peritoneal dialysis patient

**DOI:** 10.1007/s13730-022-00765-4

**Published:** 2022-12-15

**Authors:** Hironori Nakamura, Michiko Ueda, Mariko Anayama, Yasushi Makino, Masaki Nagasawa

**Affiliations:** Department of Nephrology, Shinonoi General Hospital, 666-1 Ai Shinonoi, Nagano, 388-8004 Japan

**Keywords:** Ceruloplasmin, Divalent metal transporter 1, Hypoxia-inducible factor-prolyl hydroxylase inhibitor, Transferrin saturation

## Abstract

Dysregulation in total body copper causes severe complications and excess copper can be toxic. *Divalent metal transporter 1*, *duodenal cytochrome B*, and copper transporter ATPase7A are included in the many intestinal genes transactivated by HlF-α. On July X, 2022 an 80-year-old female patient on peritoneal dialysis was prescribed roxadustat 100 mg, because darbepoetin was unable to increase hemoglobin level effectively. On the same day, icodextrin 1 L was initiated to mitigate edema. Laboratory data showed hemoglobin 9.1 g/dL, transferrin saturation 77%, copper 123 μg/dL, and iron 170 μg/dL before changing to roxadustat. The patient visited us 6 days after the change because of the appetite loss. Transferrin saturation and serum copper and iron levels increased to 90%, 170 and 203 μg/dL, respectively, which were decreased or normalized after discontinuing roxadustat and icodextrin, suggesting that even short-term roxadustat administration can influence copper levels as well as iron levels. Excess copper and iron levels during roxadustat treatment do not immediately equate with toxicity, but indicate a physiological compensation or transient imbalance of metabolism especially in patients treated with ferric citrate. Further investigation for the hypoxia-inducible factor-prolyl hydroxylase inhibitors effects on iron and copper metabolisms is needed. Determining the short-term effect of roxadustat on serum copper and iron in only this case is impossible. Therefore, further accumulation of similar cases is necessary to clarify the short-term effects of roxadustat on serum copper and iron.

## Introduction

Copper is an indispensable trace metal element and total body copper is essentially dependent on the balance between intestinal absorption, cell uptake, and bile excretion [1]. Plasma total copper levels have been reported as increased in patients with Alzheimer's disease, although causative association is still controversial [2]. Deteriorated immune function, diabetes, coronary heart disease, and osteoporosis are associated with chronic copper excess which may be harmful to our health [3]. Hypoxia-inducible factor (HIF)-prolyl hydroxylase inhibitors (HIF-PHIs) have emerged as a novel approach for renal anemia management. Several clinical studies have demonstrated that HIF-PHIs effectively corrected and maintained target hemoglobin (Hb) levels in peritoneal dialysis (PD) patients [4, 5]. Real-world study on hemodialysis patients treated with roxadustat demonstrated the effectiveness in increasing hemoglobin levels [6]. *Divalent metal transporter 1 (DMT1)*, *duodenal cytochrome B (DCYTB)*, and copper transporter ATPase copper transporting alpha (ATP7A) related to iron absorption are included in the many intestinal genes transactivated by HlF-α. Iron and copper have similar physiochemical properties and points of interaction [7], and some studies have shown that DMT1 can transport copper [8]. Recently, possible relationship between copper excess and HIF-PHI treatments was reported [9]. In this study, we report a PD case that showed increased serum levels in both copper and iron after initiating roxadustat, which were decreased or normalized after discontinuing roxadustat, suggesting that even short-term roxadustat administration can influence serum copper as well as iron levels.

## Case report

**Fig. 1:**
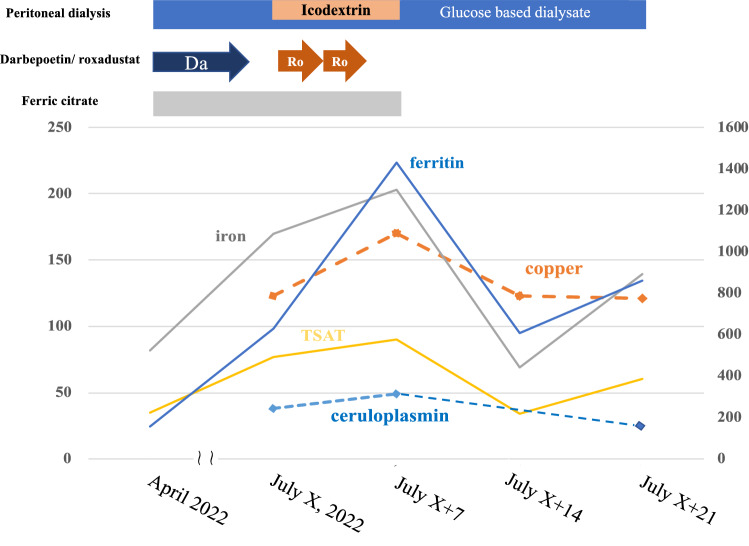
Treatment course for peritoneal dialysis and anemia management are shown. Serum copper, iron, ferritin, TSAT, and ceruloplasmin changes days after roxadustat treatment Ferritin value only, see numbers on right side of scale

An 80-year-old female patient on continuous ambulatory PD for 2 years treated with a total of 4 L of 1.5 and 2.5% glucose-based solution. The patient had an aortic valve replacement history and reduced ejection fraction. The dialysate-to-creatinine ratio measured on May 2022 was 0.80. On July X, 2022, roxadustat 100 mg, before sleep, three times a week was prescribed, because 60 μg/month of darbepoetin alfa was unable to maintain the target Hb level. On the same day, icodextrin 1L was initiated to mitigate lower extremity edema. Her body weight was 40 kg and urine volume was 300 ml per day. Hyperphosphatemia has also been treated with ferric citrate 750 mg for more than 6 months. Laboratory data showed Hb 9.1 g/dL, albumin 2.7 g/dL, ferritin 629 ng/mL, transferrin saturation (TSAT) 77%, copper 123 μg/dL (normal range 66–130 μg/dL), and iron 170 μg/dL (normal range 40–188 μg/dL) shown in Table 1. She visited us because of appetite loss and chest discomfort 6 days after the change and Hb level increased to 10.0 g/gL. The TSAT and serum copper and iron levels increased to 90%, 170 μg/dL, and 203 μg/dL, respectively, on July X + 7, 2022. Therefore, she needed to be hospitalized to reconsider anemia and chronic heart failure management. After roxadustat, ferric citrate, and icodextrin discontinuation, her appetite gradually improved after initiation of polaprezinc 150 mg, a zinc-containing compound. The TSAT as well as copper and iron levels decreased to 34%, 123 μg/dL, and 69 μg/dL, respectively, on July X + 14, 2022 (Fig. 1). The copper concentration in the efferent solution after the overnight dwell showed 3.4 μg/dL on July X + 7, 2022. Quick Auto Neo Cu (SHINO-TEST Co., Sagamihara, Japan) was used to measure serum and efferent solution copper concentration described in detail in the previous paper [9].

**Table 1:** Changes in data including hemoglobin level, albumin, aspartate aminotransferase, alanine aminotransferase, c-reactive protein, iron-related parameters, copper, zinc, ceruloplasmin, and BNP level, before and after roxadustat treatment

	April, 2022	Jun X + 2, 2022	July X, 2022	July X + 6, 2022	July X + 7, 2022	July X + 14, 2022	July X + 21, 2022
Hemoglobin (11.6–14.8 g/dL)	11.7	9.6	9.1	10.0	9.5	9.6	9.0
Albumin (4.1–5.1 mg/dL)	2.5	3.0	2.7	3.0	2.7	2.2	2.4
Aspartate aminotransferase (13–30 U/L)	24	23	22	21	15	13	15
Alanine aminotransferase (10–42 U/L)	10	12	11	11	10	10	11
C-reactive protein (mg/dL)	0.04	0.13	0.13	n.a	n.a	n.a	n.a
Copper (66–130 µg/dL)	n.a	n.a	122	n.a	170*	123	121
Zinc (80–160 µg/dL)	n.a	n.a	40.6	n.a	61.8	52.3	65.1
Iron (40–188 µg/dL)	82	n.a	170	n.a	203*	69	139
Ferritin (20–280 ng/mL)	156	n.a	629*	n.a	1430*	610*	861*
Transferrin saturation (%)	35	n.a	77	n.a	90	34	60
UIBC (µg/dL)	150	n.a	52	n.a	23	136	94
TIBC (µg/dL)	232	n.a	222	n.a	226	205	233
Ceruroplasmin (21–37 mg/dL)	n.a	n.a	38*	n.a	49*	n.a	32
BNP (5.8–18.4 pg/mL)	1772	2061	3881*	n.a	n.a	4722*	4130*
Body weight (kg)	41.4	40.8	43.3	41.7	41.7	42.0	42.0

Asterisk indicates above normal range. *n.a* not available, *UIBC* unsaturated iron-binding capacity, *TIBC* total iron-binding capacity, *BNP* brain natriuretic peptide

## Discussion

HIF-PHIs intervention effects on iron metabolism showed that ferritin, TSAT, and hepcidin decreased, and the total iron-binding capacity increased over a 24-week period according to several PD studies [5, 10]. However, the short-term serum changes in both iron and copper during HIF-PHI treatment are not well known. In the present case, the increase in ferritin, TSAT, copper, iron, and ceruloplasmin over 7 days after roxadustat initiation were noted. All these values decreased over 7 days after roxadustat and ferric citrate withdrawal, suggesting that even short-term treatment, two roxadustat oral administrations influence both iron and copper metabolism.

A short-term study [11] that investigates iron metabolism on hemodialysis demonstrated that roxadustat increased serum iron levels and TSAT 2 days after administration, which support our results seen on day X + 7. Then, serum iron and TSAT decreased on day 7.

In this case, the TSAT and ferritin level were already increased before initiating roxadustat, likely, due to ferric citrate treatment for more than 6 months and chronic heart failure exacerbation, and then, both values increased up to 90% and 1430 ng/mL, respectively, after initiating roxadustat. Although the patient had a tendency to overload iron, we speculated that roxadustat induced the imbalance between iron absorption and its use, which ended up in an increase in these iron-related parameters.

Hepcidin plays a pivotal role in the pathogenesis of inflammation-induced anemia. In contrast, hepcidin production increases in response to iron loading. Serum hepcidin levels are increased by inflammatory cytokines in hepatocytes. Erythropoietic action induction during ineffective erythropoiesis generates a preferential signal that results in hepcidin transcription suppression, even in the presence of excessive systemic iron [12]. Considering the data transition in this present case, the Hb level increased after roxadustat initiation, while iron and ferritin increased over 7 days, suggesting that hepcidin was decreased by roxadustat administration and iron absorption was induced at the intestine even in the short term. It is unclear whether the high serum iron levels will start to decrease by adjusting and improving iron metabolism if administration of roxadustat is continued.

The phase 2 study in Japan on patients undergoing hemodialysis reported that the treatment arm with roxadustat had the adverse events of nausea (5.2%) and vomiting (7.2%). In contrast, neither nausea nor vomiting was reported in the control arm with darbepoetin alpha. Increased iron and ferritin, in addition to appetite loss and rapid increase in hemoglobin (0.9 g/dL per 6 days), were reasons to discontinue roxadustat.

Ceruloplasmin is the main copper transport protein in the plasma and a known HIF-1 target. Both hypoxia and CuCl2 increased ceruloplasmin mRNA levels in hepatoma cells [13]. Ceruloplasmin is also a multicopper plasma protein containing ferroxidase activity necessary for converting Fe (II) to Fe (III) [14]. Close association between the change in ceruloplasmin level and roxadustat treatment was noted in this case. The aforementioned experimental results support our idea that roxadustat can increase serum copper and iron levels by raising the ceruloplasmin level.

A meta-analysis identified a significant association between high serum copper and heart failure (HF), and the authors speculated that the association between HF and high ceruloplasmin concentrations was caused by high serum copper levels [15]. In the present case, the short-term changes in serum copper and ceruloplasmin may be related to HF worsening or recovery in addition to roxadustat treatment.

The patient had spent her life at a public nursing home where she was provided every meal. Considering her meal management environment, the patient had less chance to have copper-rich food from July X to July X + 7.

This study has some limitations. Several clinical conditions, including renal failure [16] and anemia of inflammation [17], are known for reasons to increase serum copper due to elevated serum ceruloplasmin. Renal failure cannot be excluded and anemia of inflammation might be associated with copper excess.

Theoretically, PD could lead to greater depletion of elements than hemodialysis, because most elements are protein-bound, and significant peritoneal protein loss can occur [18]. It is unclear up to what extent serum copper level can be influenced by the amount of copper into efferent solution.

In conclusions, excess copper and iron levels during HIF-PHI treatment do not mean immediately toxicity, but indicate a physiological compensation or transient imbalance of copper and iron metabolism, especially in patients treated with ferric citrate. Determining the short-term effect of roxadustat on serum copper and iron in only this case is impossible. Therefore, further accumulation of similar cases is necessary to clarify the short-term effects of roxadustat on serum copper and iron.
